# Comparison of body mass index and physical activity levels of e-sportsmen

**DOI:** 10.3389/fpubh.2025.1557022

**Published:** 2025-02-19

**Authors:** Burhan Başoğlu

**Affiliations:** Department of Physical Education and Sports, Faculty of Sport Sciences, Nevşehir Hacı Bektaş Veli University, Nevşehir, Türkiye

**Keywords:** e-sports players, physical activity levels, body mass index (BMI), sedentary lifestyle, metabolic risk factors

## Abstract

**Background:**

This study examines the relationship between body mass index (BMI) and physical activity levels in e-sports players. Focusing on the sedentary lifestyle and health risks associated with prolonged sitting and low physical activity, the study highlights critical challenges within the e-sports community.

**Methods:**

Data was collected from 136 e-sports players on popular gaming platforms such as FIFA, Valorant, CS: GO and League of Legends. Physical activity levels were assessed using the International Physical Activity Questionnaire (Short Form) and participants’ health status was assessed using BMI classifications.

**Results:**

The results showed a significant inverse relationship between BMI and physical activity, with obese e-sports players having significantly lower activity levels and longer periods of sedentary behavior than their peers. Male e-sports players had higher levels of physical activity than females, reflecting biological, social and cultural factors that influence physical activity behavior.

**Discussion:**

The study highlights the need for measures such as training programs, ergonomic adjustments and awareness campaigns to increase physical activity and reduce health risks among e-sports players. These findings provide valuable insights into the health effects of e-sports participation and emphasize the importance of promoting an active lifestyle to counteract the detrimental effects of prolonged sitting and inactivity. By filling gaps in the existing literature, this study contributes to the understanding of e-sports players’ health behaviors and offers practical recommendations for promoting healthier habits in this growing population.

## 1 Introduction

In recent years, the concept of electronic sports (e-sports) has gained prominence as an area where digital gaming culture and the world of sports are merging (1). While evolving technology and digital platforms have led to the global spread of e-sports, the impact of this field on sports and health disciplines has also been discussed (2, 3). However, e-sports players’ prolonged sedentary habits, lack of physical activity and associated metabolic risk factors have remarkable effects on individual health and societal well-being (4).

Studies on the lifestyle of e-sports players show low levels of physical activity and increased body mass index (BMI), which is closely linked to problems such as sedentary lifestyle and obesity (5–7, 43). Prolonged sitting leads to low energy expenditure, reduced muscle mass and increased cardiovascular risks (8). Nevertheless, regular inclusion of daily physical activity can reduce these negative effects (9). In contrast to the well-known sports, i.e., physical sports, e-sports are more about the informational side than the physical activity (10).

Social and cultural factors play an important role in the level of physical activity of e-sports players. In particular, gender differences have shown that female e-sports players have lower levels of physical activity participation than men (4, 11, 12). Prolonged sitting is not only limited to the effects on individual health, but also leads to a spread of sedentary lifestyles in society.

The low physical activity of e-sports players leads to a lower metabolic threshold. This leads to a decline in athletic performance as the efficiency of the energy cycle is reduced (13–16). However, the literature largely confirms that interventions aimed at increasing physical activity lead to a significant improvement in metabolic health (17–19).

The level of physical activity and lifestyle of e-sports players are associated not only with effects on metabolic health, but also on cognitive and motor performance. Motor learning plays an important role in improving individuals’ reflexes and strategic movements (20, 21). e-sports players use cognitive and motor skills simultaneously to improve in-game decision making and reaction times (22). However, increasing physical activity can not only have positive effects on metabolic health, but also contribute to a more efficient development of motor skills (23). The positive effects of regular physical activity on cognitive performance and motor skills are widely confirmed in the literature. Himmelstein et al. (24), for example, emphasize that improving the cognitive and motor skills of e-sports players not only improves individual performance, but also coordination within the team. In addition, the use of visual and auditory feedback in the training process of e-sports players optimizes the motor learning process (25). In this context, when investigating the effects on BMI and physical activity levels of e-sports players, it should not be forgotten that interventions to develop motor skills are part of a holistic approach to health and performance.

The aim of this study was to comprehensively investigate the relationship between body mass index (BMI) and physical activity levels of e-sports players and to assess metabolic risk factors in this context. The evolving digital gaming culture and the increasing popularity of e-sports have significantly influenced people’s lifestyle choices and physical activity habits. In particular, the prolonged sitting and low physical activity of professional e-sports players can lead to health problems such as obesity, cardiovascular disease and metabolic syndrome. In this context, the main objective of this study is to make a new and original contribution to the literature on the impact of e-sports on health by analyzing biological and behavioral indicators related to the lifestyle of e-sports players. By jointly evaluating the BMI and physical activity of e-sports players, the study highlights the dynamics between these two important variables.

There are only a limited number of studies in the literature on the long sitting times and low energy consumption of e-sports players. However, the present study addresses these issues from a broader perspective and provides a more comprehensive understanding of the health status of e-sports players. In addition, the study also examines the effects of gender and social factors on the physical activity levels of e-sports players, filling a knowledge gap in this area. It is expected that the results will inform future intervention programs to protect individual health and promote healthier lifestyle for e-sports players. In this regard, the research has the potential to have practical implications not only for the academic literature, but also for e-sport players and health professionals. In this context, the following problem statements (PS) were investigated as part of the study.

PS 1. Does the body mass index of e-sports players increase, their physical activity level decrease and the time spent sitting increase?

PS 2. Do male e-sports players have a higher level of physical activity compared to female e-sports players?

PS 3. Does the habit of sitting for long periods of time have a negative effect on the overall METs of e-sports players?

## 2 Method

This study was conducted using a descriptive design to compare the body mass index (BMI) and physical activity levels of e-sports players. As part of the study, the “International Physical Activity Questionnaire (Short Form)” was used to determine the physical activity level of e-sports players.

### 2.1 Working group

A total of 136 e-sports players took part in this study. The participants were selected from four different industries that are popular in the world of e-sports: CS: GO, FIFA, LoL and Valorant. Participants was selected using the random sampling method and participation was voluntary. Basic demographic characteristics of the e-sports players participating in the study such as age, gender, weight, height, e-sports industry, weekly e-sports playtime and body mass index (BMI) were recorded and included in the analysis. This information formed an important basis for more detailed evaluations in relation to the general scope of the study and the research questions. The data to be used for the study was collected between November 15 and December 15, 2024 via Google Forms using the random sampling method.

### 2.2 Statistical analysis

In this study, descriptive and comparative statistical methods were used to analyse the data. Descriptive analysis was used to determine the basic characteristics of the participants and calculate frequency distributions. As it was found that the data did not have a normal distribution, non-parametric tests were preferred. The Kruskal-Wallis H test was used to assess differences between body mass index (BMI) and physical activity level, and the Mann–Whitney U test was used for pairwise comparisons between groups. These analyses were conducted to examine the differences and relationships between physical activity level and BMI of the e-sports players.

### 2.3 Data collection tool

In the data analysis, the International Physical Activity Questionnaire (Short Form) (26) was used to assess the level of physical activity of the participants. Separate MET minutes/week scores were calculated for walking (3.3 METs), moderate activity (4.0 METs) and vigorous activity (8.0 METs) when scoring the questionnaire. The participants’ total physical activity score was calculated by adding the METs of all activities. Based on these scores, participants were divided into three different categories according to their physical activity level: Inactive (<600 MET-min/week), minimally active (600–3,000 MET-min/week) and highly active (>3,000 MET-min/week). This classification served as the basis for determining the physical activity level and for comparisons between the groups.

## 3 Results

This study delves into several critical dimensions of the demographic, behavioral, and health profiles of e-sports players. It offers a detailed analysis of age and gender distributions, revealing a male majority and a high concentration of participants in the 20–21 age bracket. The study also examines e-sports engagement trends, identifying FIFA as the most favored game and noting substantial variations in weekly playtimes among players. Furthermore, the data underscores a significant link between BMI and physical activity levels, with obese players showing notably lower engagement in vigorous and moderate-to-vigorous activities and reporting longer sitting durations compared to their healthy and overweight peers. Clear gender-based disparities emerge as well, with males demonstrating higher levels of physical activity than females. Finally, the findings highlight a progressive decline in total MET values as BMI increases, painting a comprehensive picture of the physical activity and lifestyle patterns common among e-sports players.

According to Table 1, the age distribution of e-sports players is highest in the 20–21 age group at 41.9%. With a total of 57 people, this group represented the age group with the highest participation in the study. In terms of gender distribution, the majority of participants were men at 84.6%, while the proportion of female participants was 15.4%.

**Table 1:** Demographic information of the e-sports players participating in the study.

Age of participants	*N*	%
18–19 years old	17	12.5
20–21 years old	57	41.9
22–23 years old	7	5.1
24–25 years old	28	20.6
26–27 years old	16	11.8
28 years and older	11	8.1
Gender		
Woman	21	15.4
Male	115	84.6
Total	136	100.0

Table 2 shows that the highest participation rate among the e-sports industries was recorded for FIFA with 33.8%. This was followed by Valorant with 27.9%, LoL (League of Legends) with 25.0% and CS: GO (Counter-Strike: Global Offensive) with 13.2%. These results show that respondents tend to favor popular games such as FIFA and Valorant.

**Table 2:** Information about e-sports of the e-sports players participating in the research.

Branches (Games)	*N*	%
CS GO	18	13.2
FIFA	46	33.8
LoL	34	25.0
Valorant	38	27.9
Weekly e-sports playing time		
5 h and below	48	35.3
7 h	8	5.9
8 h	41	30.1
9 h	7	5.1
10 h or more	32	23.5

When analyzing the weekly e-sports playing time, the proportion of participants who play 5 h or less is the highest at 35.3%. In second place are those who play for 8 h, at 30.1%. Furthermore, those who play for 10 h or more are at 23.5%, while the other periods of 7 h and 9 h are at 5.9 and 5.1%, respectively. These results show that the majority of participants play their weekly e-sports activity in the range of 5–8 h.

According to Table 3, significant differences were observed in physical activity levels as BMI increased. In particular, it was determined that the obese group (30–39.9 kg/m^2^) had significantly lower scores in the levels of vigorous physical activity and moderate-to-vigorous physical activity compared to the other groups (*p* < 0.05). Individuals in the obese group (25–29.9 kg/m^2^) showed a higher mean at the level of vigorous physical activity than healthy individuals (18.5–24.9 kg/m^2^) (B > C). In terms of sitting time, obese individuals sat significantly longer than the other groups (A < B < C, *p* < 0.05). In addition, when total METs were taken into account, obese individuals had a significantly lower physical activity level than obese individuals (C < B, *p* < 0.05). These findings clearly show that as BMI increases, physical activity decreases and sitting time increases.

**Table 3:** Comparison of body mass index and physical activity scores of e-sportsmen.

	VKİ	*N*	Mean	S.d.	*X* ^2^	*p*
Vigorous physical activity (MET-min/week)	^A^18. 5 kg/m^2^ – 24. 9 kg/m^2^ [Healthy]	59	2242.7	2034.6	16.500	0.000* B > C
	^B^25 kg/m^2^ – 29. 9 kg/m^2^ [Fatty]	47	2713.0	2047.4		
	^C^30 kg/m^2^ ile 39. 9 kg/m^2^ [Obesity]	30	1204.0	2026.5		
Moderate to vigorous physical activity (MET-min/week)	^A^18. 5 kg/m^2^ – 24. 9 kg/m^2^ [Healthy]	59	611.5	616.6	12.419	0.002* C < A.B
	^B^25 kg/m^2^ – 29. 9 kg/m^2^ [Fatty]	47	1115.3	1407.3		
	^C^30 kg/m^2^ ile 39. 9 kg/m^2^ [Obesity]	30	213.3	366.8		
Walking (MET-min/week)	^A^18. 5 kg/m^2^ – 24. 9 kg/m^2^ [Healthy]	59	1187.4	758.4	5.652	0.059
	^B^25 kg/m^2^ – 29. 9 kg/m^2^ [Fatty]	47	979.8	912.4		
	^C^30 kg/m^2^ ile 39. 9 kg/m^2^ [Obesity]	30	1068.1	1372.2		
Sitting time (min)	^A^18. 5 kg/m^2^ – 24. 9 kg/m^2^ [Healthy]	59	643.4	137.3	25.195	0.000* A > B < C A < C
	^B^25 kg/m^2^ – 29. 9 kg/m^2^ [Fatty]	47	522.6	193.8		
	^C^30 kg/m^2^ ile 39. 9 kg/m^2^ [Obesity]	30	807.0	278.5		
Total MET	^A^18. 5 kg/m^2^ – 24. 9 kg/m^2^ [Healthy]	59	4685.1	2512.5	12.762	0.002* C < B
	^B^25 kg/m^2^ – 29. 9 kg/m^2^ [Fatty]	47	5330.7	2814.3		
	^C^30 kg/m^2^ ile 39. 9 kg/m^2^ [Obesity]	30	3292.4	3052.5		

*X*^2^Kruskal Wallis Test, * = *p* < 0.50.

According to Table 4, significant differences were found between the genders in the values for vigorous physical activity and moderate to vigorous physical activity. The male participants had significantly higher values than the females at the vigorous physical activity level (males: 2355.7 MET-min/week; females: 1192.3 MET-min/week, *p* = 0.022). Men also had a higher mean value for moderate to vigorous physical activity than women (men: 671.8 MET-min/week; women: 840.0 MET-min/week, *p* = 0.012). Although there was no significant difference in walking and sitting times, men had higher walking times than women and similar sitting times (*p* > 0.05). Although there was no statistically significant difference between the sexes in total MET values (*p* = 0.061), men had higher mean MET values than women (men: 3435.0 MET-min/week; women: 1137.9 MET-min/week). These results suggest that men generally have higher levels of physical activity than women.

**Table 4:** Comparison of body mass index and physical activity scores of e-sportsmen in terms of gender.

	Gender	*N*	Mean	S.d.	*z*	*p*
Vigorous physical activity (MET-min/week)	Woman	21	1192.3	1556.2	−2.287	0.022*
	Male	115	2355.7	2139.3		
Moderate to vigorous physical activity (MET-min/week)	Woman	21	840.0	546.2	−2.512	0.012*
	Male	115	671.8	1052.4		
Walking (MET-min/week)	Woman	21	823.4	619.6	−2.512	0.339
	Male	115	1137.9	1017.3		
Sitting time (min)	Woman	21	579.2	129.1	−0.955	0.638
	Male	115	648.4	232.0		
Total MET	Woman	21	3435.0	1915.8	−1.870	0.061
	Male	115	4813.9	2917.5		

^*^p < 0.05.

## 4 Discussion

This study investigated the relationship between body mass index (BMI) and physical activity in e-sports players. A decrease in physical activity and an increase in sedentary behavior were observed with increasing BMI. These findings provide important clues for assessing the health status of e-sports players and increasing their physical activity levels.

The findings showed that e-sports players with obesity had significantly lower MET values than people with a healthy BMI during both vigorous and moderate to vigorous physical activity. Similarly, obese individuals had significantly lower total MET values than the other groups (Table 3). This suggests that obese individuals are limited in their physical activity and are more likely to lead a sedentary lifestyle. These findings support previous studies in the literature on the relationship between obesity and low physical activity. For example, Rey-López et al. (27) reported that increasing BMI limits physical activity and promotes sedentary behavior. They also emphasized that this inverse relationship between obesity and physical activity triggers metabolic health problems (27). The studies by Remmers et al. and Pandey et al. (28, 29) examined the relationship between increasing BMI and decreasing physical activity and reported that obese individuals are reluctant to participate in physical activity due to low energy levels. This highlights the influence of metabolic and psychological factors on physical activity in obese individuals (28, 29). The WHO (30) emphasized that increasing physical activity plays a crucial role in improving quality of life and preventing metabolic diseases, especially in obese individuals. Similarly, del Pozo-Cruz et al. (31) found that a sedentary lifestyle increases the risk of cardiovascular disease in obese individuals. Specifically for e-sports players, DiFrancisco-Donoghue et al. (32) reported that the influence of BMI on physical activity levels is more pronounced in e-sports players due to their sedentary lifestyle. This finding once again shows that physical activity should be increased, especially in e-sports players. Furthermore, it has been reported that e-sports players who spend long periods of time sitting not only negatively affect body weight and cardiovascular disease risk, but also posture and muscle activity (33–36).

In this study, men were found to have significantly higher MET values during intense physical activity compared to women (Table 4). The higher intensity of physical activity in men compared to women shows the influence of biological, social and cultural factors in shaping physical activity behavior. In particular, it is assumed that men take more time for physical activity, while women are limited in this area due to their daily commitments. These results are consistent with the findings from the literature. Ekelund et al. (37) reported that men have higher levels of physical activity compared to women, which is particularly evident during strenuous physical activity. Similarly, Dumith et al. (38) emphasized that the physical activity habits of men are associated with higher energy expenditure than those of women. These gender differences have been linked to social and environmental barriers to physical activity. For example, Bauman et al. (39) reported that the factors that prevent women from participating in physical activity are related to factors such as childcare, housework and lack of social support. In parallel, Trost et al. (40) reported that women’s lower participation in physical activity is often due to individual factors such as lack of motivation and lack of time. In addition, Hagger and Chatzisarantis (41) found that gender differences are related to self-efficacy perceptions in relation to physical activity. To increase women’s participation in physical activity, it is recommended to strengthen social support mechanisms and develop women-specific programs. The WHO report (2022), for example, emphasizes that increasing physical activity is not only an individual responsibility, but also a social responsibility.

In this study, it was found that e-sports players are sedentary for longer and especially obese individuals are sedentary for longer compared to other groups (Tables 3, 4). As e-sports activities inherently promote a sedentary lifestyle, this suggests the need for strategies to increase physical activity in e-sports players. The tendency of e-sport players to spend long periods of time sitting has been widely addressed in the existing literature. Booth et al. (42) reported that sedentary lifestyles are prevalent in screen-based activities such as digital gaming and e-sports, which can lead to health problems such as metabolic syndrome, cardiovascular disease and diabetes. In addition, Lam et al. (43) emphasized that the sedentary lifestyle of e-sports players can have negative effects not only on physical health but also on mental health.

Rezende et al. (44), in a study looking at the effects of sedentary behavior on general public health, found that the increased risks associated with sedentary behavior can be avoided through regular physical activity. Similarly, Owen et al. (45) reported that prolonged sitting limits energy expenditure and that individuals who sit for long periods during activities such as e-sports have a higher risk of developing metabolic syndrome.

The need to increase the physical activity of e-sports players has also been addressed in terms of athletic performance. Many studies have found that regular physical activity can increase e-sports players’ reflex speed and in-game performance (1, 5, 43, 46–48). These findings suggest that physical activity is not only important for health, but also for professional success.

Developing strategies to overcome the lack of physical activity is important on both an individual and societal level. For example, many studies have pointed out that interventions against physical inactivity should be planned not only at the individual level but also at the societal level (49–53). The WHO report (2019) emphasizes that increasing physical activity is crucial for global health goals and recommends the expansion of programs, especially for sedentary people (54). In summary, the findings of this study clearly indicate that educational programs, opportunities for physical activity and awareness campaigns to increase physical activity among e-sports players should be expanded.

## 5 Conclusion

This study demonstrated the relationship between BMI and physical activity in e-sports players and showed that as BMI increased, physical activity decreased and sitting time increased. Notably, obese e-sports players had significantly lower MET levels than healthy individuals during both vigorous and moderate-intensity physical activity, highlighting the critical role of physical activity in the treatment of obesity. Furthermore, when gender differences were analyzed, men were found to have higher levels of physical activity than women. This difference was linked to social and environmental barriers as well as biological and cultural factors. More social support and specific programs need to be developed to increase women’s participation in physical activity.

A sedentary lifestyle is widespread among e-sports players. E-sports players who tend to sit for long periods of time can develop health problems such as obesity, metabolic syndrome and cardiovascular disease. This not only has a negative impact on physical health, but also on mental health. However, it has been reported that regular physical activity can improve e-sports players’ reflex speed, in-game performance and overall health. These findings suggest that physical activity is not only important for health, but also for professional success.

To summarize, it is necessary to develop strategies to increase the physical activity of e-sports players. Educational programs, awareness raising and ergonomic adjustments can be an important part of these strategies. In addition, societal and individual initiatives to reduce sedentary lifestyles have the potential to improve the health and performance levels of e-sports players.

Regular exercise programs and breaks to reduce sitting time should be encouraged to increase the physical activity of e-sports players. Specific support mechanisms and programs should be developed to increase the participation of female e-sports players in physical activity. In addition, awareness campaigns and ergonomic adjustments can reduce the negative effects of sedentary lifestyles. These approaches will improve both the health and performance of female e-sports players.

### 5.1 Limitations and future research

This study provides valuable insights into the physical activity and health profiles of esports players. However, it is important to acknowledge certain limitations that may impact the scope and interpretation of the findings.

Firstly, the sample predominantly consists of male e-sports participants (84.6%), which limits the study’s ability to represent the female e-sports population adequately. This lack of diversity highlights a potential issue in the generalizability of the findings to a broader demographic, particularly female players.

Furthermore, the use of self-reported data collected through an online questionnaire introduces the risk of reporting bias. Participants might have overestimated or underestimated their physical activity levels and health metrics, which could influence the accuracy of the results. Incorporating objective measures, such as wearable fitness trackers, in future research could help validate self-reported data and enhance the reliability of the findings. Another limitation lies in the study’s focus on specific e-sports environments and gaming disciplines, including FIFA, Valorant, League of Legends, and CS: GO. As a result, the conclusions drawn may not necessarily be applicable to all types of e-sports athletes or gaming genres. Future research should strive to include a broader range of gaming disciplines, exploring variations in physical activity and health profiles across diverse e-sports contexts.

Acknowledging these limitations is essential for guiding future studies and ensuring the cautious interpretation of the findings. Addressing these concerns in subsequent research will contribute to a more comprehensive understanding of the health and physical activity behaviors of e-sports players, ultimately advancing the field.

## Data Availability

The raw data supporting the conclusions of this article will be made available by the authors, upon reasonable request. Requests to access these datasets should be directed to basoglu.burhan61@gmail.com.
